# Inflammation and atrial fibrillation

**DOI:** 10.1002/joa3.12984

**Published:** 2024-01-03

**Authors:** Yasushi Mukai

**Affiliations:** ^1^ Division of Cardiology, Fukuoka Red Cross Hospital Fukuoka Japan

**Keywords:** antiinflammatory therapy, atrial fibrillation, cytokines, inflammation, post‐operative AF

Editorial comment to “Subacute postoperative atrial fibrillation after heart surgery: Incidence and predictive factors in cardiac rehabilitation.”^1^

Numerous investigations have indicated that inflammatory mechanisms play important roles in the pathogenesis and progression of atrial fibrillation (AF).^2^, ^3^, ^4^ Inflammatory responses in the atrial wall may lead to changes in electrophysiological property and structural changes of atrial myocardial tissue with fibrosis, which lead to occurrence and persistence of AF. Roles of inflammatory cells/cytokines may be important both in acute and chronic mechanisms in the various disease course of AF.

## 1 INFLAMMATION AND POSTOPERATIVE AF

Postoperative atrial fibrillation (POAF) occurs in up to 15%–60% of cardiac surgery and is associated with increased risks of various morbidities, longer hospital stay, and mortality. The occurrence of POAF may be largely influenced by the systemic and local/pericardial inflammatory responses due to operative invasion.

Neutrophil‐to‐lymphocyte ratio (NLR) is an easily available inflammatory index and is proposed to be a risk marker of AF.^5^ In the present study by Rizza et al., the authors focused on subacute POAF in cardiac rehabilitation (CR) phase and its predisposing factors. They found that age, mitral valve procedures, acute POAF, and pre‐operative NLR were associated with occurrence of subacute POAF.^1^ The authors concluded that recognition of arrhythmic predictors might be useful to stratify patients' risk in order to tailor treatment strategies and optimize their prognosis during postoperative period. Interestingly, postoperative (T1) NLR or CRP was not significantly correlated to the occurrence of sPOAF in multiple logistic regression analysis. It is conceivable that baseline state of the immune system may have a significant impact to the occurrence of sPOAF. Another point of interest is that the history of AF is significant but only weakly correlated to the occurrence of sPOAF, a morbidity in the specific clinical situation.

## 2 ROLE OF INFLAMMATION IN THE PATHOGENESIS OF AF

Incidence of AF increases in the presence of classical risk factors such as advanced age, hypertension, diabetes, and heart failure. As mediating mechanisms, inflammation, and oxidative stress are considered to be important in the long‐term disease course of AF. It is also reported that incidence of AF is larger in those with chronic inflammatory diseases such as collagen disease and inflammatory bowel disease. Clinical and experimental studies indicated elevated serum inflammatory biomarkers and the expression of inflammatory markers or cytokines/growth factors in cardiac tissues in AF subjects. Inflammatory responses in the atrial wall may lead to increased oxidative stress, myocardial apoptosis, fibrosis, gap junction modulation, and intracellular calcium‐handling abnormalities, which result in the development of arrhythmogenic substrate and AF. For example, white blood cell counts or ratio, serum C‐reactive protein (CRP), tumor necrosis factor‐alpha (TNF‐α), monocyte chemoattractant protein‐1 (MCP‐1), transforming growth factor (TGF‐β) and Interleukins (ILs) were studied and supposed to be involved in the atrial tissue remodeling and development of AF. Local inflammatory responses in atrial wall or in adjacent tissues may directly cause AF. These specific mechanisms may be involved in pericarditis, myocarditis, and POAF, as is in the present study.

## 3 ANTI‐INFLAMMATORY STRATEGY FOR THE MANAGEMENT OF AF

It can be hypothesized that anti‐inflammatory drugs or intervention may be useful to prevent and treat AF. Various anti‐inflammatory therapies for AF have been already studied and reported.^2^, ^3^, ^4^ It has been reported that corticosteroids may be useful in reducing recurrence of AF after catheter ablation as well as POAF. A short‐term use of colchicine reduced AF burden after catheter ablation as well as POAF. Some reports suggested that statins may be beneficial in reducing POAF via anti‐inflammatory actions. Many other anti‐inflammatory drugs and strategies have been reported to be effective to inhibit AF occurrence in specific settings. In addition to the management of classical risk factors and specific AF therapies such as anti‐arrhythmic drugs or catheter ablation, control of systemic and atrial tissue inflammation should be taken into consideration in order to totally overcome the pathophysiology of AF.

## CONFLICT OF INTEREST STATEMENT

The authors declare no conflict of interests for this article.
